# A Novel Single-Layer Microfluidic Device for Dynamic Stimulation, Culture, and Imaging of Mammalian Cells

**DOI:** 10.3390/bios15070427

**Published:** 2025-07-03

**Authors:** Adil Mustafa, Antonella La Regina, Elisa Pedone, Ahmet Erten, Lucia Marucci

**Affiliations:** 1 School of Engineering Mathematics and Technology, University of Bristol, Bristol BS8 1TW, UK; elisa.pedone@bristol.ac.uk; 2 School of Cellular and Molecular Medicine, University of Bristol, Bristol BS8 1TD, UK; 3 Department of Electronics and Communication Engineering, Istanbul Technical University, Istanbul 34469, Turkey; aerten@itu.edu.tr

**Keywords:** microfluidics, micromixers, mammalian cells, single layer, PDMS

## Abstract

The possibility of tightly controlling the cellular microenvironment within microfluidic devices represents an important step toward precision analysis of cellular phenotypes in vitro. Microfluidic platforms that allow both long-term mammalian cell culture and dynamic modulation of the culture environment can support quantitative studies of cells’ responses to drugs. Here, we report the design and testing of a novel microfluidic device of simple production (single Polydimethylsiloxane layer), which integrates a micromixer with vacuum-assisted cell loading for long-term mammalian cell culture and dynamic mixing of four different culture media. Finite element modeling was used to predict flow rates and device dimensions to achieve diffusion-based fluid mixing. The device showed efficient mixing and dynamic exchange of media in the cell-trapping chambers, and viability of mammalian cells cultured for long-term in the device. This work represents the first attempt to integrate single-layer microfluidic mixing devices with vacuum-assisted cell-loading systems for mammalian cell culture and dynamic stimulation.

## 1. Introduction

Culturing cells in vitro is of paramount importance in molecular biology in order to study, for example, cellular processes such as cell–cell interactions, responses to external stimuli (physical or chemical), and acquisition of genetic mutations over time [1,2,3]. Traditional methods of cell culturing (i.e., in dishes) do not allow for spatiotemporal control of the cellular environment and are therefore limited when it is important to study phenotypic changes upon dynamic cell perturbations. Microfluidic devices find their advantage in using low sample volumes while allowing precise control over the cell culture environment and mimicking of physiological conditions [4,5,6,7,8]. Dynamic drug stimulation and media mixing, as well as long-term media perfusion, can be of great importance for mammalian cell biology studies. This is often achieved by integrating within microfluidic devices active and passive micromixers [9,10,11], and using multiple layers (which come with the cost of sophisticated mold preparation) and/or separated devices for multiplexer input control and cell growth, respectively [12,13]. Dettinger and colleagues [14] reported an automated microfluidic device with 48 independent chambers for both adherent and suspension mammalian cell cultures in rapidly changing media conditions; mixing was implemented on-chip, but this device is still quite complex to fabricate as it includes valves and multiple layers. Jaberi et al. designed a microfluidic device which generated mechanical and chemical stresses to facilitate 3D cell cultures in microchambers [15]. The device requires a specialized fabrication process where micropillars need to be embedded into microchambers to keep the cells in position over long time-lapse experiments. A more recent approach towards automating microfluidic cell culture platforms was reported by Pitingolo et al. [16], where the authors reported a device embedding storage, control, and cell culture units. Kane and colleagues [17] reported a multilayer microfluidic device consisting of a stratified array of 96 microfluidic chips integrated within a robotics system, enabling long-term culture of mammalian cells as well as automation of seeding, feeding, and other cell culture processes. This device requires quite complex fabrication and assembly processes.

Single-layer microfluidic platforms have been proposed to simplify the fabrication process and to facilitate cell loading [18,19]. Usually, such devices allow for long-term mammalian cell culture but lack the ability to dynamically mix/switch between more than two different media.

Regarding applications, there has been an increasing interest in employing microfluidic devices in high throughput single-cell studies [20,21,22,23,24,25], parallelized drug screening [12], temporal modulation of inputs [26], and cell proliferation and differentiation studies [27,28]. Regarding the latter, the most commonly used device is one proposed by Kolnik and colleagues [26]; it is a multilayer microfluidic device with a built-in function generator to create chaotic mixing between two different cell culture media. The device implements vacuum-assisted loading of cells in individual trapping chambers to protect them from shear stress and has been successfully used in most of the external feedback control work regarding mammalian cells [29,30,31,32]. One key limitation is that cells can be perturbed and controlled with only two inputs.

**Figure 1:**
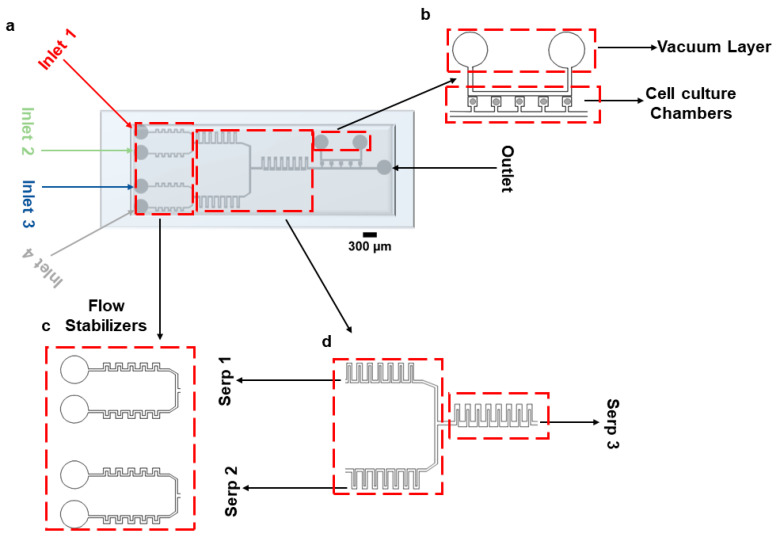
Microfluidic device design. (**a**) Graphical representation of the device bonded on a glass slide. (**b**) Vacuum layer and cell culture chambers. (**c**) Inlet part of the device with flow stabilizing serpentines. (**d**) Mixing serpentines 1 and 2, which facilitate diffusive mixing of cell media and drugs; serpentine 3 enables diffusive mixing of culture media coming from serpentines 1 and 2.

In this study, we present a novel Polydimethylsiloxane (PDMS), single-layer microfluidic device that can be employed to culture mammalian cells over the long-term while perfusing them with combinations of up to four cell culture media (possibly enriched with drugs). The device embeds a cell-loading part, a diffusive mixing part, and five cell culture chambers adjoined to the perfusion channel (Figure 1). As in [26], vacuum-assisted cell loading is employed. Media are provided using four inlets; each of them is automatically regulated by software-controlled syringe pumps and connected to flow stabilizers and mixing serpentine structures. We show, via finite element analysis-based simulations and with experiments, that the device allows for mixing of different inputs and dynamic exchange of the different inputs provided to cells. The device also allows for long-term culturing of different mammalian cell lines.

The device addresses key challenges in microfluidic cell culturing by incorporating cell-trapping chambers specifically designed to minimize shear and compressive stress, promoting sustained cell viability without the need for external mechanical isolation. The compact single-layer design enhances optical accessibility, reduces fabrication complexity, and allows for easy integration into standard biological workflows, making it particularly suitable for widespread use in biomedical and translational research.

Our new device has specific advantages such as single-layer fabrication, diffusion-based mixing of four different inputs, and easy cell loading. To our knowledge, this is the first study to use a single-layer microfluidic device with an integrated vacuum-assisted cell-loading mechanism. It should be possible to easily modify the device to increase its drug-screening capability, for example, by modifying the number of inlets and serpentines.

## 2. Materials and Methods

### 2.1. Device Design

To facilitate reagent delivery and biochemical stimulation, a rectangular serpentine micromixer was incorporated into the device connecting it to the vacuum-assisted cell culture chambers. While more advanced micromixer architectures exist in the literature, the primary aim of this work was to maintain a single-layer, lithographically defined device that is easy to fabricate, reproducible, and suitable for widespread adoption in standard biological laboratories. Although this structure does not represent a novel advance in micromixer engineering, it provides sufficient mixing efficiency for our intended application, which involves the perfusion of growth media and the stimulation of mammalian cells. The device consists of 5 consecutive cell culture chambers (with a footprint of 360 µm × 360 µm, Figure 1a). The ‘opening’ that connects the chambers to the main channel is 200 µm × 200 µm. The height of the channel is 50 µm. Cells are loaded using a vacuum pump connected to the vacuum layer running parallel to the main channel, as shown in Figure 1b. Figure 1c,d represent the flow stabilizers and serpentine structures for fluid stabilization and mixing, respectively. The distance between the vacuum channels and the trapping chambers is 80 µm. A combination of different cell culture media/drugs can be infused through the device using four inlets, as shown in Figure 1. Inlets 1 and 2 come together in the top serpentine (Serp 1) structure, and Inlets 3 and 4 come together in the bottom serpentine (Serp 2) for mixing purposes. A larger serpentine structure (Serp 3) is connected at the junction of Serp 1 and Serp 2 for further mixing of the fluids coming from them. The dimensions of the serpentine, the number of serpentines required to achieve complete mixing, the fluid flow rates, and the number of cell culture chambers were chosen given results from COMSOL 5.6 simulations. Figure S1 reports the steps required for device fabrication.

### 2.2. Mixing Experiments and Imaging

**Figure 2:**
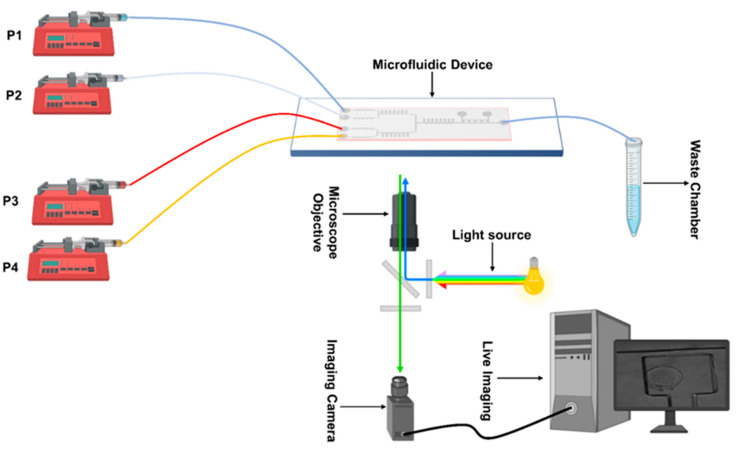
A schematic of the experimental setup for cell culturing and imaging on-chip.

**Figure 3:**
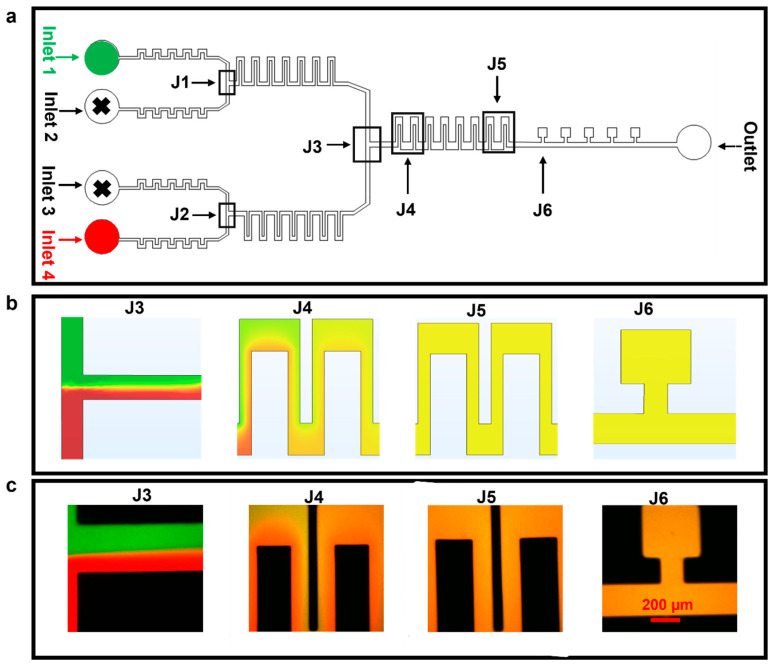
Mixing of two different fluids. The flow rate used for these experiments was 50 µL h^−1^. (**a**) Microfluidic device with highlighted parts. (**b**) COMSOL simulation depicting diffusion-based mixing of two fluids using inlets 1 and 4. (**c**) Experimental results for static mixing using Atto 488 and Rhodamine B dyes at 50 µL h^−1^.

**Figure 4:**
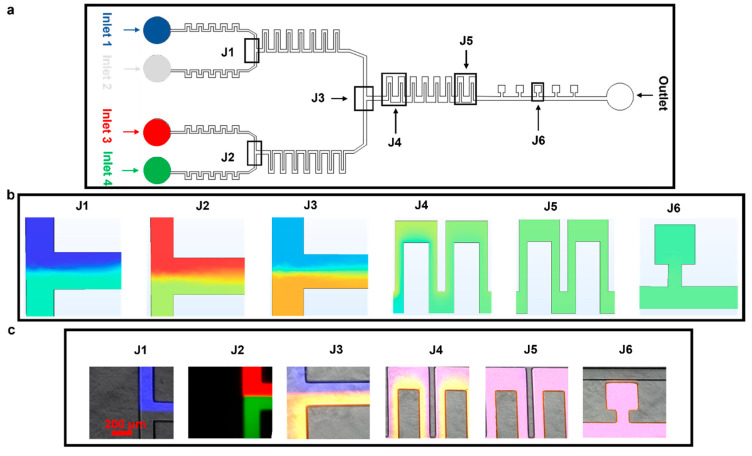
Mixing of four different fluids. The experiments were designed to test the ability of the device to achieve mixing using serpentine structures. The flow rate at all the inlets was set at 50 µL h^−1^. **(a**) Microfluidic device highlighting different parts. (**b**) COMSOL simulation depicting diffusion-based mixing of four fluids using inlets 1–4. (**c**) Experimental results for static mixing using Atto 488, Rhodamine B, Atto 647, and PBS at 50 µL h^−1^.

**Figure 5:**
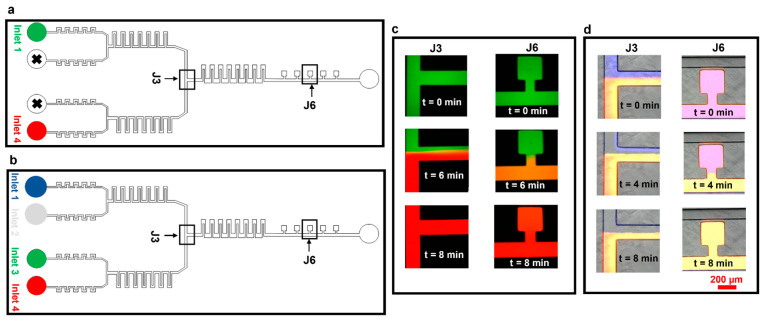
Media exchange experiments using Atto 488 and Rhodamine B. The experiments were designed to test the dynamic media exchange ability of the device by using two inlets. (**a**) Schematic of the device highlighting inlets being used for experiments in panel ‘c’. (**b**) Schematic of the device highlighting inlets being used for experiments in panel ‘d’. (**c**) Experimental results of media exchange using two dyes. (**d**) Experimental results of media exchange using three dyes and PBS.

For media delivery, we used syringe pumps (AL-1000, World Precision Instruments, Sarasota, FL, USA), as shown in Figure 2. A customized Matlab code was used to run the pumps and change the flow rates during dynamic mixing and media exchange experiments. The imaging setup consisted of a Leica LASX live cell imaging workstation on a DMi8 inverted fluorescence microscope (Leica Microsystems, Wetzlar, Germany). An Andor iXON 897 Ultra (Andor Technology, Belfast, UK) back integrated camera was used for imaging. Images collected to test media exchange and mixing were recorded by using an objective lens with a magnification of 10×; cell viability experiments’ images were collected using a 20× magnification. For experiments in Figures 3–5 and in Supplementary Figure S3, media were enriched with fluorescence dyes Atto 488 (41051-1MG-F), Atto 647 (97875-1MG-F), Rhodamine B CAS-81-88-9, and Phosphate-buffer saline (D8537-500 ML), all obtained from Sigma Aldrich (St. Louis, MS, USA), and used at a 10 µM concentration.

**Figure 6:**
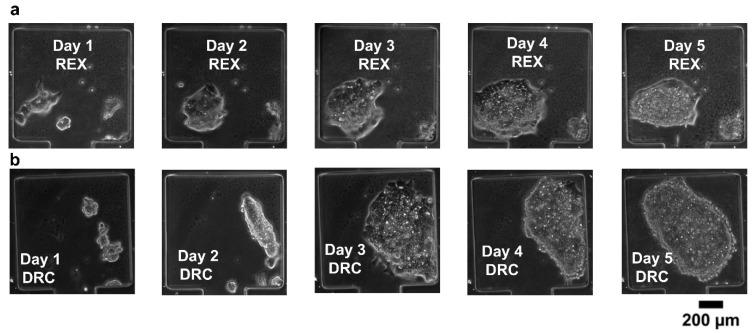
(**a**,**b**) Cell viability tests for REX-dGFP2 (REX, **a**) and MiR-290-mCherry/miR-302-eGFP (DRC, **b**) mouse embryonic stem cell lines.

The media exchange and mixing experiments in Figures 3–5 were 30 min long with images recorded every 2 min, while the experiment in Figure S3 was 145 min long with images recorded every 5 min. For cell viability experiments (Figure 6), the microscopy images were collected every 24 h.

Of note, the experiments where the fluid concentrations were changed dynamically required balancing of the flow rates in the main junction; this is because the total flow rate at inlets 1 and 2 needs to be kept equal to the total flow rate at inlets 3 and 4. Such balancing avoids any back flow from inlets 3 and 4 towards inlets 1 and 2. For example, if the flow rate at inlet 1 and inlet 2 is 50 µL h^−1^ in each (100 µL h^−1^ in total), the total flow rate at inlets 3 and 4 should also be equal to 100 µL h^−1^. Thus, in experiments where the flow rate at one of the inlets was changed dynamically, the flow rate in the other inlet(s) was adjusted.

The chip wetting process for the experiments where only two of the four inlets were used was handled carefully. Initially, wetting was carried out by keeping inlets 2 and 3 open; the pumps connected to inlets 1 and 4 were started at high flow rates (i.e., between 6–10 mL h^−1^) to fill the entire device. This resulted in fluids from inlets 1 and 4 flowing out of open inlets 2 and 3. Afterwards, these inlets were sealed using knotted tubing. This procedure minimizes air bubble generation.

### 2.3. Diffusion and Flow Modeling

Fabrication of microfluidic devices requires resources and time not readily available. Hence, it is of utmost importance that device design is optimized using all the available tools before the fabrication process is started. Computational fluid dynamics helps in the design optimization of microfluidic devices. To verify the operating parameters of our device, we used COMSOL 5.6 Multiphysics software. The device design generated by AutoCAD was imported into COMSOL 5.6 for the purpose of simulation. The computational fluid dynamics (CFDs) model was based on solving fluid flow by using a laminar form of the Navier–Stokes equation and modeling the transport mechanism using mass transport equations. The fluid flow was modeled using ‘laminar flow’ built-in Physics while the transport mechanism was solved using the ‘transport of diluted species’ interface. Simulations were set up using a fully developed flow boundary condition at the inlets to accurately model the laminar flow regime; the outlet boundary condition was set to zero pressure. The dyes were used at the same concentration as the experiments in all simulations to validate the experiments. The diffusion coefficient values for Rhodamine B and Atto 488 were used to simulate diffusive mass transport in the device. The governing equations, fluid velocity magnitude, and shear stress in serpentine structures, connecting channels, and cell culture chambers are provided in Supporting Information S2.

### 2.4. Cell Culture and Device Loading

For viability experiments (Figure 6), two mouse Embryonic Stem Cell (mESC) lines (REX-dGFP2 [33] and MiR-290-mCherry/miR-302-eGFP [34]) were cultured in serum-based media. The microfluidic device was pre-wetted with 1 mL of Dulbecco’s modified Eagle’s medium at room temperature via a 2.5 mL syringe. Before loading in the chip, mESCs were cultured on gelatinized tissue culture dishes at 37 °C in a 5% CO_2_ humidified incubator in Dulbecco’s modified Eagle’s medium (DMEM) supplemented with 15% fetal bovine serum (Sigma), non-essential amino acids, L-glutamine, sodium pyruvate, Penicillin-Streptomycin, 2-mercaptoethanol, and 10 ng/mL LIF (Peprotech; 250-02). Once the mESCs in p10 culture dishes reached confluency around 2.2 × 10^6^ cells, they were detached using 3 mL Gibco trypsin-EDTA (0.5%). A total of 1 mL of detached cells suspended in trypsin was collected in a falcon and centrifuged at 1200 RPM for 5 min. The supernatant was removed using a micropipette and the cells were then resuspended in 100 µL of the cell culture media. The cells were then loaded into a 2.5 mL syringe, avoiding any bubbles. The cell loading into the microfluidic device was carried out in two steps. First, the chip was wetted by using the cell culture media. In the next step, the syringe with cells was attached to the outlet and cells were infused into the chip. Once the channel was filled with the cells, a vacuum pump at a pressure of −80 kPa connected to the vacuum layer was switched on to aspirate the cells into the chambers and to remove any air in the chambers. Once loaded, the device was infused with fresh media to clear the excess cells.

### 2.5. Cell Culture and Device Loading for the Open Loop Experiment

An open loop experiment was performed with Rex-dGFP2 cells [33]. mESCs were grown on gelatin-coated dishes and cultured in serum-free 2i medium, composed of NDiff227 basal medium and supplemented with Chiron-99021, hereafter called CH, which inhibits glycogen synthesis kinase-3 GSK3; PD0325901 at 3 µM, hereafter called PD, which inhibits mitogen-activated protein MEK1/2; and CGP77675, which inhibits SRC, at 1 µM [35]. mESCs were kept for 2–3 passages (around 6–10 days) in 2i medium before running the experiment. The device was pre-filled with 2i medium before cell loading. A confluent p60 petri dish was used for the experiment with 3.2 × 10^6^ cells. Media from the petri dish of mESCs were collected and centrifuged at 1200 rpm for 5 min, whereas the cells still attached were trypsinized for 5 min at room temperature. After 5 min, the cells were filtered with a 40 µm and centrifuged at 1200 rpm for 5 min. Pelleted cells were resuspended in 50 µL of medium 2i and loaded using a 2 mL syringe into the main channel from the outlet port. The device was perfused with 2i medium at 150 µL h^−1^ overnight inside the incubator at 37 °C and 5% CO_2_, before starting external feedback control experiments.

Before starting the experiment, syringes (10 mL) were filled with NDiff (syringes 1 and 4), 2i + LIF (Leukemia inhibitory factor -LIF-, which activates Janus kinases-Signal transducers and activators of transcription JAK/STAT and mitogen-activated protein kinases MAPK at 1 µM) and 1 µM of Atto 647 (syringe 2), and 2i and 1 µM of sulphorodamine (syringe 3). The media were delivered via four external pumps at a flow rate of 150 µL h^−1^ each. Pump 1 (P1) and pump 4 (P4) were loaded with NDiff medium, pump 2 (P2) with 2i + LIF medium, and pump 3 (P3) with 2i. The experiment was designed to combine pumps switching simultaneously and to ensure continuous flow at junction J3. The four pumps were associated with the relative inlet (i.e., pump 1/inlet1, pump 2/inlet2, pump 3/inlet3, pump 4/inlet4) and simultaneously switched on for delivering the following: 2i medium (P1–P3), 2i + LIF (P2–P4), and NDiff (P1–P4). Once the microfluidic device was connected and located to the microscopy stage maintained at 37 °C and 5% CO_2_, the experiment started.

Images were acquired using a microscope with a sampling time of 1 h in phase contrast (PH) and Green Fluorescence Intensity (GFP). To segment cells and quantify fluorescence, we relied on a thresholding method we previously developed [36].

## 3. Results

### 3.1. Diffusion Mixing Experiments

Firstly, we tested, via simulations and with experiments, the device performance in mixing media from the different inlets (first just two, in Figure 3, and then four, in Figure 4). The finite element method was used to model the fluid flow and the mixing in the microfluidic device. For experimental tests, we mixed two media containing Atto 488 (green dye) or Rhodamine B (red dye) and infused with flow rates of between 50–100 µL h^−1^ in inlets 1 and 4; inlets 2 and 3 were not used in these experiments and were closed (Figure 3a). It was observed that, at 50 µL h^−1^, the two fluids flowed side by side in the section of the chip known as J3 and mixed completely as they reached the section of the chip denoted as J5 and, ultimately, the cell culture chambers of J6 (simulation and experiment in Figure 3b and c, respectively). Of note, mixing at flow rates higher than 100 µL h^−1^ might be possible by increasing the number of serpentine structures.

The device performance was further tested by mixing four different fluids; this was achieved by combining fluids in 2 × 2 configurations, as shown in Figure 4a. We kept the flow rate at 50 µL h^−1^ at all four inlets. The fluids from inlet 1 (Atto 647, blue dye) and inlet 2 (phosphate-buffered silane, PBS) were combined in the top junction and were mixed via J1; similarly, the fluids from inlets 3 (Rhodamine B, red dye) and 4 (Atto 488, green dye) came together at the bottom section and were mixed via J2 (Figure 4b,c, simulation and experiment, respectively).

As observed when mixing two fluids, there was a thorough mixing of the four fluids in J5 and in the cell culture chambers at J6 (Figure 4b,c, simulation and experiment, respectively). Differences in the mixed media color measured in experiments/simulations are because the colorless media used in experiments could not be represented in COMSOL, where a light blue color was instead used and a light green color represented the Atto 488 dye.

We then tested our device for effective media exchange. We first set up experiments using only two inlets, infused with two fluorescence dye solutions (Rhodamine B (red) and Atto 488 (green), Figure 5a,c). Initially, the pump controlling the syringe with the green dye was set at 50 µL h^−1^ and the pump controlling the red dye-containing syringe was switched off. In this case, the whole microfluidic device, including cell culture chambers, was filled with the green dye (t = 0 min, Figure 5c). We then (t = 4 min) switched off the pump infusing the green dye and switched on the pump with the red dye at 50 µL h^−1^. At t = 6 min, the red dye started to take over the green one in the junction, taking over the perfusion channel and the cell culture chambers at t = 8 min. We also confirmed robust media exchange, mixing, and flow stability in longer experiments (2 h and 40 min, Supplementary Information S3).

The device performance was further assessed by setting up media exchange experiments using all four inlets (Figure 5b,d). Experiments were performed by connecting inlets 1–4 to the pumps infusing dye solutions Atto 647 (blue), PBS (colorless), Rhodamine B (green), and Atto 488 (red), respectively. Initially, all four solutions were infused at 50 µL h^−1^ (time t = 0 min, Figure 5d). The pump infusing the blue dye (inlet 1) was then completely switched off and the flow rate at the pump infusing PBS (inlet 2) was increased to 100 µL h^−1^ to balance the flow rates at inlets 3 and 4. At t = 4 min, PBS started taking over the blue dye at section J3; complete media exchange was obtained at t = 8 min (Figure 5d, sections J3 and J6).

### 3.2. Cell Viability Experiments

Long-term cell viability is an essential requirement in systems biology. To aid this, we carefully considered the mechanical stress factors commonly encountered in cell culture platforms. Stress-induced cell damage, particularly from fluid shear and compressive forces, poses a significant barrier to maintaining viable cultures over extended periods. To mitigate these effects, we designed the cell-trapping chambers with dimensions of 360 × 360 µm and a narrow neck region measuring 120 µm in length. This configuration was specifically chosen to shield the cells from excessive mechanical stress, thereby providing a more stable microenvironment conducive to long-term viability. We then performed cell viability experiments to assess the suitability of our microfluidic device for long-term mammalian cell cultures. REX-dGFP2 [33] and MiR-290-mCherry/miR-302-eGFP (referred to here as DRC) [34] mouse embryonic stem cell (mESCs) lines were used for this purpose. The microfluidic devices loaded with cells were incubated at 37 °C and perfused with continuous media flow (syringe pump at 50 µL h^−1)^. The results in Figure 6 show that both cell lines remain healthy and expand in our microfluidic device for at least 5 days.

**Figure 7:**
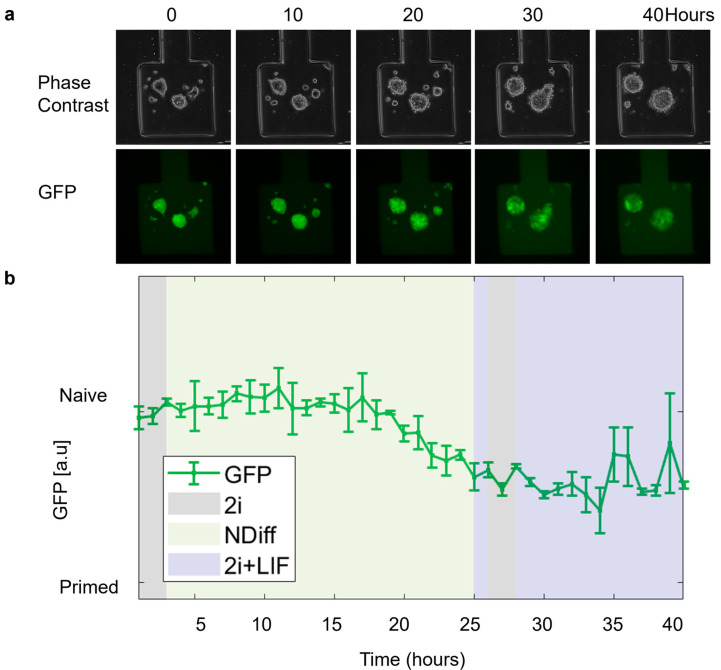
mESCs open loop experiment. (**a**) Phase contrast and Green Fluorescence Protein (GFP) images of REX-dGFP2 cells acquired at the indicated time points. (**b**) Green Fluorescence Protein (GFP) intensity (measured every hour) of the cell segmentation mask over time calculated using ChipSeg [36]. For the first 3 h (initialization), cells were kept in 2i medium to measure maximum expression (100%). The green line denotes the average % of GFP (Green Fluorescence Protein) across the chambers (*n* = 2). Purple, gray, and yellow regions indicated delivery of different inputs (2i + LIF and 2i and NDiff, respectively).

We performed further experiments to demonstrate the possibility of culturing and perturbing mESCs in the device while imaging them via time-lapse microscopy. In REX-dGFP2 cells, treatment with 2i (i.e., basal NDiff medium with the addition of Chiron-99021 and MEK inhibitor PD0325901) or 2i + LIF (2i with the addition of LIF) can increase Rex1 and, thus, GFP expression, pushing cells to a naïve pluripotency state; and vice versa, drug removal can make cells transition to a primed state with decreased Rex1 expression. As in Figure 7, cells showed a healthy morphology during the whole time-lapse, and the GFP signal (quantified using image segmentation) showed the expected response to media change, although with some delay.

## 4. Conclusions

Exposing cultured cells to a specific medium for a determined amount of time is of utmost importance for many biomedical and biological studies [37,38]. It is therefore necessary to have a controlled and rapid mechanism of media exchange for microfluidic devices that are designed and fabricated for such experiments.

In this study, we present a novel single-layer microfluidic device that combines a passive microfluidic micromixing mechanism with vacuum-assisted cell loading for long-term mammalian cell culturing. Our device allows for cell culturing with reduced shear stress and dynamical cell stimulation with combinations of four different media, which can be automatically delivered to cells via computer-controlled pumps. The mixing of the fluids is achieved by rectangular serpentine structures at fluid flow velocities in the µL h^−1^ range. Finite element method simulations and experiments showed successful fluid mixing and media exchange in cell-trapping chambers at flow velocities of 50−100 µL h^−1^, avoiding shear stressing of cells. Long-term cell culture (5 days) of two different mammalian cell lines, in a fully controllable culture environment, was achieved. Our device, as compared to previously reported ones, is easier to fabricate and use, being single layer. The design (available at https://github.com/LM-group/Single-layer-microfluidic-device-.git (accessed on 23 June 2025)) could be modified for different types of cell lines and applications.

Compared to existing platforms, our approach offers a unique balance between simplicity, scalability, and functionality, making it highly suitable for routine use in biomedical research, drug screening, and personalized diagnostics. Future work may extend this design for co-culture applications, integration with biosensors, or automation in high-throughput settings.

We foresee interest in using this and comparable devices in systems biology applications, where it is important to measure cell responses upon dynamic drug stimulation and derive/distinguish mathematical models of the systems [39], and in synthetic biology, both to quantitatively characterize responses to inputs in synthetic gene networks [40] and to implement external feedback control using microfluidics/microscopy platforms [29,30,31,36].

## Data Availability

Raw data of the experiments are available at https://github.com/LM-group/Single-layer-microfluidic-device-.git, accessed on 23 June 2025. Codes used for the experiments and the analysis are available upon request.

## Supplementary Materials

The following supporting information can be downloaded at: https://www.mdpi.com/article/10.3390/bios15070427/s1, Figure S1: Device fabrication protocol. (a) Four-inch silicon wafer. (b) Spin coating SU-8 2050 negative photoresist on a four-inch silicon wafer. (c) Exposure of spin-coated silicon wafer using laser source. (d) Removal of excess negative photoresist using SU-8 developer solution. (e) PDMS replica molding. (f) Final fabricated device bonded on glass slide. The CAD file is added in the supplementary information and is also freely available at GitHub. Figure S2: (a) Flow velocity (in m/s) in serpentine, flow channel, and cell-trapping chambers. (b) Shear rate (in s^−1^) in serpentine, flow channel, and cell-trapping chambers. Figure S3: Media exchange experiments for extended time at 50 µL h^−1^. (a) Microfluidic device design with highlighted parts. (b) Images of the junction indicated in (a). At the start of the experiment, we have only green dye, and at t = 135 min the inlet with green dye is switched off. The red dye starts to take over the junction, and at t = 145 min it completely takes over the junction. (c) Images of the first cell culture chamber. At t = 145 min, it completely takes over the cell culture chamber.

## References

[1] Torino S., Corrado B., Iodice M., Coppola G. (2018). PDMS-Based Microfluidic Devices for Cell Culture. Inventions.

[2] Bergmann S., Steinert M. (2015). From Single Cells to Engineered and Explanted Tissues: New Perspectives in Bacterial Infection Biology. Int. Rev. Cell Mol. Biol..

[3] Ruppen J., Cortes-Dericks L., Marconi E., Karoubi G., Schmid R.A., Peng R., Marti T.M., Guenat O.T. (2014). A microfluidic platform for chemoresistive testing of multicellular pleural cancer spheroids. Lab Chip.

[4] Mehling M., Tay S. (2014). Microfluidic cell culture. Curr. Opin. Biotechnol..

[5] Lenshof A., Laurell T. (2010). Continuous separation of cells and particles in microfluidic systems. Chem. Soc. Rev..

[6] Rothbauer M., Zirath H., Ertl P. (2018). Recent advances in microfluidic technologies for cell-to-cell interaction studies. Lab Chip.

[7] Halldorsson S., Lucumi E., Gomez-Sjoberg R., Fleming R.M.T. (2015). Advantages and challenges of microfluidic cell culture in polydimethylsiloxane devices. Biosens. Bioelectron..

[8] Matsuura K., Hashioka S., Takata K. (2024). Sorting differentiated mammalian cells using deterministic lateral displacement microfluidic devices. Anal. Sci..

[9] Ward K., Fan Z.H. (2015). Mixing in microfluidic devices and enhancement methods. J. Micromech. Microeng..

[10] Broeren S., Pereira I.F., Wang T., den Toonder J., Wang Y. (2023). On-demand microfluidic mixing by actuating integrated magnetic microwalls. Lab Chip.

[11] Chatani T., Shiraishi S., Miyazako H., Onoe H., Hori Y. (2023). L-2L ladder digital-to-analogue converter for dynamics generation of chemical concentrations. R. Soc. Open Sci..

[12] Schuster B., Junkin M., Kashaf S.S., Romero-Calvo I., Kirby K., Matthews J., Weber C.R., Rzhetsky A., White K.P., Tay S. (2020). Automated microfluidic platform for dynamic and combinatorial drug screening of tumor organoids. Nat. Commun..

[13] Romero C.A., Lozano L.M., Perfecto Y., Santa Cruz F.J., Garcia-Varela R., Chairez I. (2025). Multi-cell lines culturing system utilizing microfluidic three-dimensional additive manufactured polymeric scaffolds with controlled pumping. Microchem. J..

[14] Dettinger P., Frank T., Etzrodt M., Ahmed N., Reimann A., Trenzinger C., Loeffler D., Kokkaliaris K.D., Schroeder T., Tay S. (2018). Automated Microfluidic System for Dynamic Stimulation and Tracking of Single Cells. Anal. Chem..

[15] Jaberi A., Monemian Esfahani A., Aghabaglou F., Park J.S., Ndao S., Tamayol A., Yang R. (2020). Microfluidic Systems with Embedded Cell Culture Chambers for High-Throughput Biological Assays. ACS Appl. Bio Mater..

[16] Pitingolo G., He Y., Huang B., Wang L., Shi J., Chen Y. (2020). An automatic cell culture platform for differentiation of human induced pluripotent stem cells. Microelectron. Eng..

[17] Kane K.I.W., Moreno E.L., Hachi S., Walter M., Jarazo J., Oliveira M.A.P., Hankemeier T., Vulto P., Schwamborn J.C., Thoma M. (2019). Automated microfluidic cell culture of stem cell derived dopaminergic neurons. Sci. Rep..

[18] Wang L., Ni X.F., Luo C.X., Zhang Z.L., Pang D.W., Chen Y. (2009). Self-loading and cell culture in one layer microfluidic devices. Biomed. Microdevices.

[19] Luo C., Zhu X., Yu T., Luo X., Ouyang Q., Ji H., Chen Y. (2008). A fast cell loading and high-throughput microfluidic system for long-term cell culture in zero-flow environments. Biotechnol. Bioeng..

[20] Klein A.M., Mazutis L., Akartuna I., Tallapragada N., Veres A., Li V., Peshkin L., Weitz D.A., Kirschner M.W. (2015). Droplet barcoding for single-cell transcriptomics applied to embryonic stem cells. Cell.

[21] Macosko E.Z., Basu A., Satija R., Nemesh J., Shekhar K., Goldman M., Tirosh I., Bialas A.R., Kamitaki N., Martersteck E.M. (2015). Highly Parallel Genome-wide Expression Profiling of Individual Cells Using Nanoliter Droplets. Cell.

[22] Mustafa A., Pedone E., Marucci L., Moschou D., Lorenzo M.D. (2022). A flow-through microfluidic chip for continuous dielectrophoretic separation of viable and non-viable human T-cells. Electrophoresis.

[23] ur Rehman A., Zabibah R.S., Kharratian S., Mustafa A. (2022). Microfluidic Device for the Separation of Non-Metastatic (MCF-7) and Non-Tumor (MCF-10A) Breast Cancer Cells Using AC Dielectrophoresis. JoVE.

[24] Wu W., Zhang S., Zhang T., Mu Y. (2021). Immobilized Droplet Arrays in Thermosetting Oil for Dynamic Proteolytic Assays of Single Cells. ACS Appl. Mater. Interfaces.

[25] Gharib G., Butun I., Muganli Z., Kozalak G., Namli I., Sarraf S.S., Ahmadi V.E., Toyran E., van Wijnen A.J., Kosar A. (2022). Biomedical Applications of Microfluidic Devices: A Review. Biosensors.

[26] Kolnik M., Tsimring L.S., Hasty J. (2012). Vacuum-assisted cell loading enables shear-free mammalian microfluidic culture. Lab Chip.

[27] Nocera G.M., Viscido G., Criscuolo S., Brillante S., Carbone F., Staiano L., Carrella S., di Bernardo D. (2022). The VersaLive platform enables microfluidic mammalian cell culture for versatile applications. Commun. Biol..

[28] Yazdian Kashani S., Keshavarz Moraveji M., Bonakdar S. (2021). Computational and experimental studies of a cell-imprinted-based integrated microfluidic device for biomedical applications. Sci. Rep..

[29] Postiglione L., Napolitano S., Pedone E., Rocca D.L., Aulicino F., Santorelli M., Tumaini B., Marucci L., di Bernardo D. (2018). Regulation of Gene Expression and Signaling Pathway Activity in Mammalian Cells by Automated Microfluidics Feedback Control. ACS Synth. Biol..

[30] Pedone E., de Cesare I., Zamora-Chimal C.G., Haener D., Postiglione L., La Regina A., Shannon B., Savery N.J., Grierson C.S., di Bernardo M. (2021). Cheetah: A Computational Toolkit for Cybergenetic Control. ACS Synth. Biol..

[31] Pedone E., Postiglione L., Aulicino F., Rocca D.L., Montes-Olivas S., Khazim M., di Bernardo D., Pia Cosma M., Marucci L. (2019). A tunable dual-input system for on-demand dynamic gene expression regulation. Nat. Commun..

[32] Khazim M., Pedone E., Postiglione L., di Bernardo D., Marucci L. (2021). A microfluidic/microscopy-based platform for on-chip controlled gene expression in mammalian cells. Synth. Gene Circuits.

[33] Wray J., Kalkan T., Gomez-Lopez S., Eckardt D., Cook A., Kemler R., Smith A. (2011). Inhibition of glycogen synthase kinase-3 alleviates Tcf3 repression of the pluripotency network and increases embryonic stem cell resistance to differentiation. Nat. Cell Biol..

[34] Parchem R.J., Ye J., Judson R.L., LaRussa M.F., Krishnakumar R., Blelloch A., Oldham M.C., Blelloch R. (2014). Two miRNA clusters reveal alternative paths in late-stage reprogramming. Cell Stem Cell.

[35] Ying Q.L., Wray J., Nichols J., Batlle-Morera L., Doble B., Woodgett J., Cohen P., Smith A. (2008). The ground state of embryonic stem cell self-renewal. Nature.

[36] de Cesare I., Zamora-Chimal C.G., Postiglione L., Khazim M., Pedone E., Shannon B., Fiore G., Perrino G., Napolitano S., di Bernardo D. (2021). ChipSeg: An Automatic Tool to Segment Bacterial and Mammalian Cells Cultured in Microfluidic Devices. ACS Omega.

[37] An D., Kim K., Kim J. (2014). Microfluidic System Based High Throughput Drug Screening System for Curcumin/TRAIL Combinational Chemotherapy in Human Prostate Cancer PC3 Cells. Biomol. Ther..

[38] Ruzycka M., Cimpan M.R., Rios-Mondragon I., Grudzinski I.P. (2019). Microfluidics for studying metastatic patterns of lung cancer. J. Nanobiotechnol..

[39] Marucci L. (2017). Nanog Dynamics in Mouse Embryonic Stem Cells: Results from Systems Biology Approaches. Stem Cells Int..

[40] Marucci L., Santini S., di Bernardo M., di Bernardo D. (2011). Derivation, identification and validation of a computational model of a novel synthetic regulatory network in yeast. J. Math. Biol..

