# Supplementary material for: A Novel Single-Layer Microfluidic Device for Dynamic Stimulation, Culture, and Imaging of Mammalian Cells
Source: Biosensors (Basel). 2025 Jul 3;15(7):427. doi: 10.3390/bios15070427 (PMC12293461; doi:10.3390/bios15070427)
Supplement: Supplementary file 1 [file biosensors-15-00427-s001.zip › biosensors-3637839-supplementary.pdf]

biosensors

Supplementary

A Novel Single-Layer Microfluidic Device for Dynamic Stimulation, Culture, and Imaging of Mammalian Cells

Adil Mustafa [1,2,*,†], Antonella La Regina [1,2,*,†], Elisa Pedone [1,2], Ahmet Erten [3] and Lucia Marucci [1,2,*]

[1] School of Engineering Mathematics and Technology, University of Bristol, Bristol BS8 1TW, UK; elisa.pedone@bristol.ac.uk
[2] School of Cellular and Molecular Medicine, University of Bristol, Bristol BS8 1TD, UK
[3] Department of Electronics and Communication Engineering, Istanbul Technical University, Istanbul 34469, Turkey; aerten@itu.edu.tr
* Correspondence: a.mustafa@bolton.ac.uk (A.M.); a.laregina@bristol.ac.uk (A.L.R.); lucia.marucci@bristol.ac.uk (L.M.)
† These authors contributed equally to this work.

S1: Device Fabrication

The microfluidic device reported in this study was fabricated using maskless photolithography (Figure S1). Computer-aided design software (CAD) AutoCAD was used to design the input files; a negative photoresist SU-8 2050 (MicroChemicals GMBH, Stuttgart, Germany) was spin-coated on a four-inch silicon wafer (Figure S1a) at 3000 rpm for 30 seconds to achieve a height of 50 µm, as shown in Figure S1b. A pre-exposure bake was performed in two steps by placing the wafer directly on to the hot plate at 65 °C for 2 minutes and then at 95 °C for 9 minutes.

The baked silicon wafer was then exposed using a Heidelberg laser writer 'µPG101' (Heidelberg Instruments Mikrotechnik GmbH) using 30 mW of power at 10% (Figure S1c). A post-exposure baking step was performed first at 65 °C for 1 minute and then at 95 °C for 7 minutes. The exposed wafer was then immersed in the SU-8 developer solution (MicroChemicals GMBH, Stuttgart, Germany) to remove the access photoresist. The structure was obtained in the development step (Figure S1d), which we refer to as the 'mother mold'. To further enhance the adhesion of structures to the wafer, a hard baking was done by placing the mother mold directly on to the hot plate at 200 °C for 10 minutes.

The final device was obtained by pouring PDMS on the mother mold with a base-to-curing-agent ratio of 10:1 (Figure S1e). A degassing step was performed to remove the air bubbles by placing the mold with PDMS in a desiccator for 25 minutes. After degassing, the mother mold was placed in an oven at 70 °C for 120 minutes. The baked PDMS mold was autoclaved to achieve sterilization. The individual chips were realized by bonding the PDMS devices on a microscope glass slide (75 mm × 25 mm) using oxygen plasma at 100% power, as shown in Figure S1f.

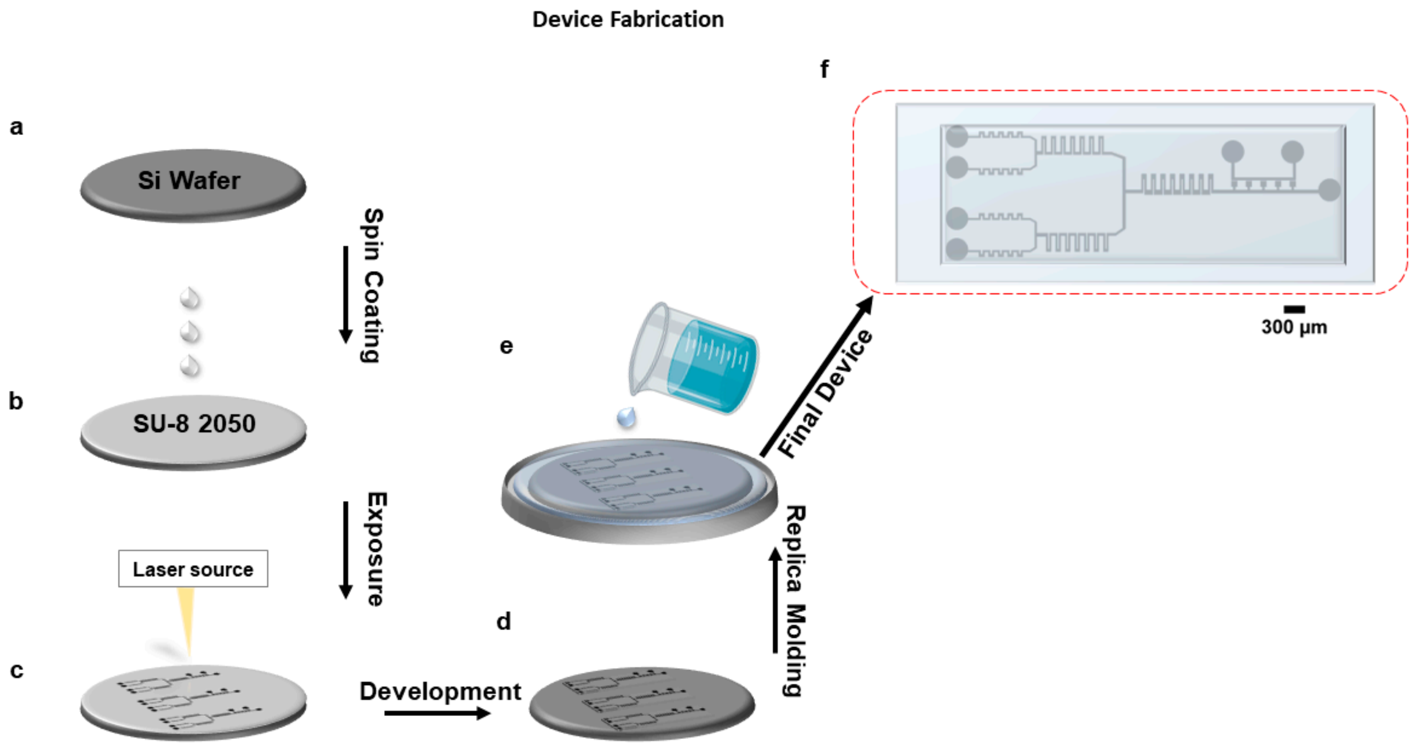

Figure S1. Device fabrication protocol. (a) Four-inch silicon wafer. (b) Spin coating SU-8 2050 negative photoresist on a four-inch silicon wafer. (c) Exposure of spin-coated silicon wafer using laser source. (d) Removal of excess negative photoresist using SU-8 developer solution. (e) PDMS replica molding. (f) Final fabricated device bonded on glass slide. The CAD file is added in the supplementary information and is also freely available at GitHub.

S2: FEM Analysis of Shear Stress

Numerical simulations based on finite element methods are routinely used to aid the development of microfluidic devices by verifying the design and operating parameters. The governing equations for our model are given as follows:

$$\rho \frac{\partial u}{\partial t} + \rho(\boldsymbol{u}_{fluid}.\nabla)\,\boldsymbol{u}_{fluid} = \nabla.[-p\mathbf{I} + \mathbf{K}] + \boldsymbol{F} \quad (1)$$

$$\rho\nabla.\boldsymbol{u}_{fluid} = 0 \quad (2)$$

$$\nabla.\boldsymbol{j}_i + \boldsymbol{u}.\nabla c_i = \boldsymbol{R}_i \quad (3)$$

$$\boldsymbol{j}_i = -\boldsymbol{D}_i\nabla_i \quad (4)$$

Time-dependent simulations were set up to mimic the experiments. Flow velocities in the main channel and cell traps were calculated using the General Laminar Flow. The magnitude of flow velocity and shear rate in serpentines, flow channels, and cell culture chambers are depicted in Figure S2. The flow velocity magnitude and the shear rate have their maximum values in the serpentines and minimum in the cell culture chambers. This ensures the claim that cells trapped in cell culture chambers are not exposed to excessive fluid shear stress, enabling them to proliferate.

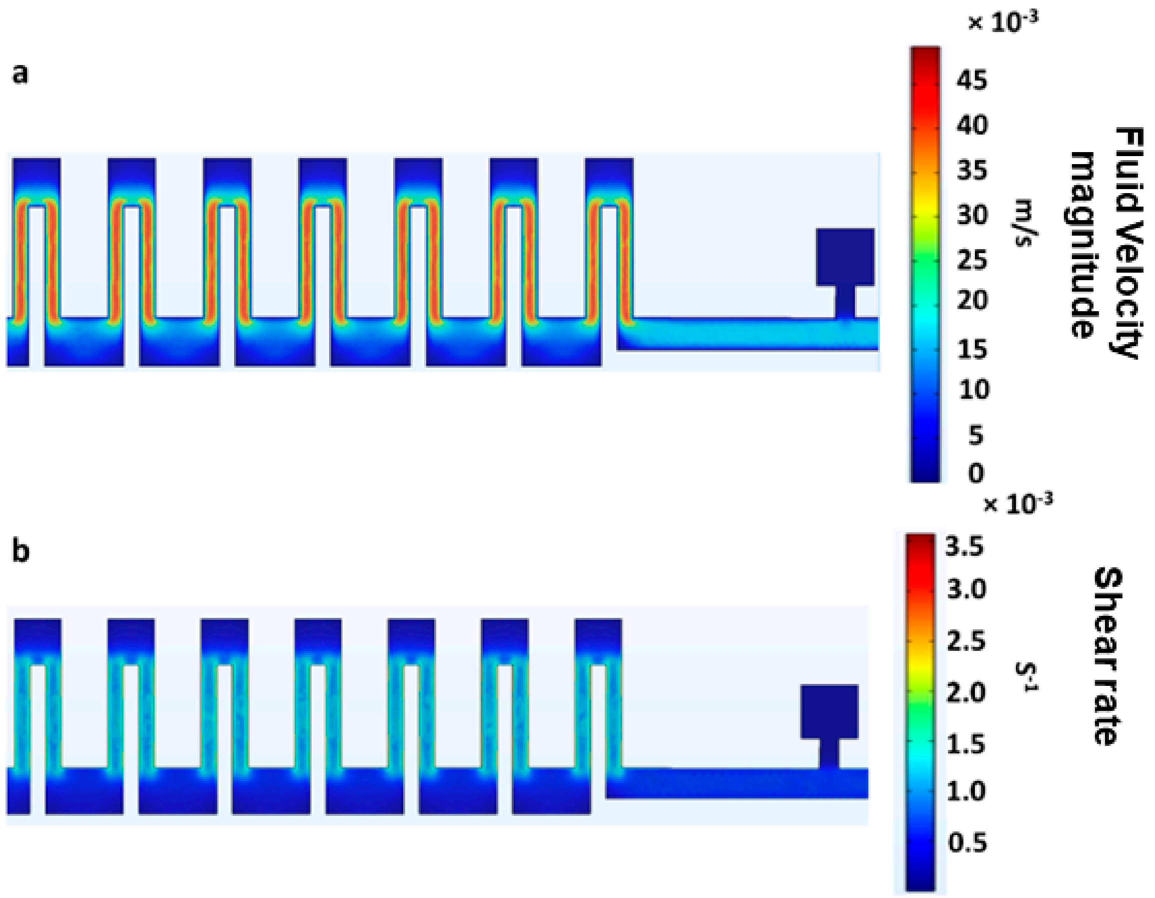

Figure S2. (a) Flow velocity (in m/s) in serpentine, flow channel, and cell-trapping chambers. (b) Shear rate (in s^{-1}) in serpentine, flow channel, and cell-trapping chambers.

S3: Media exchange experiments

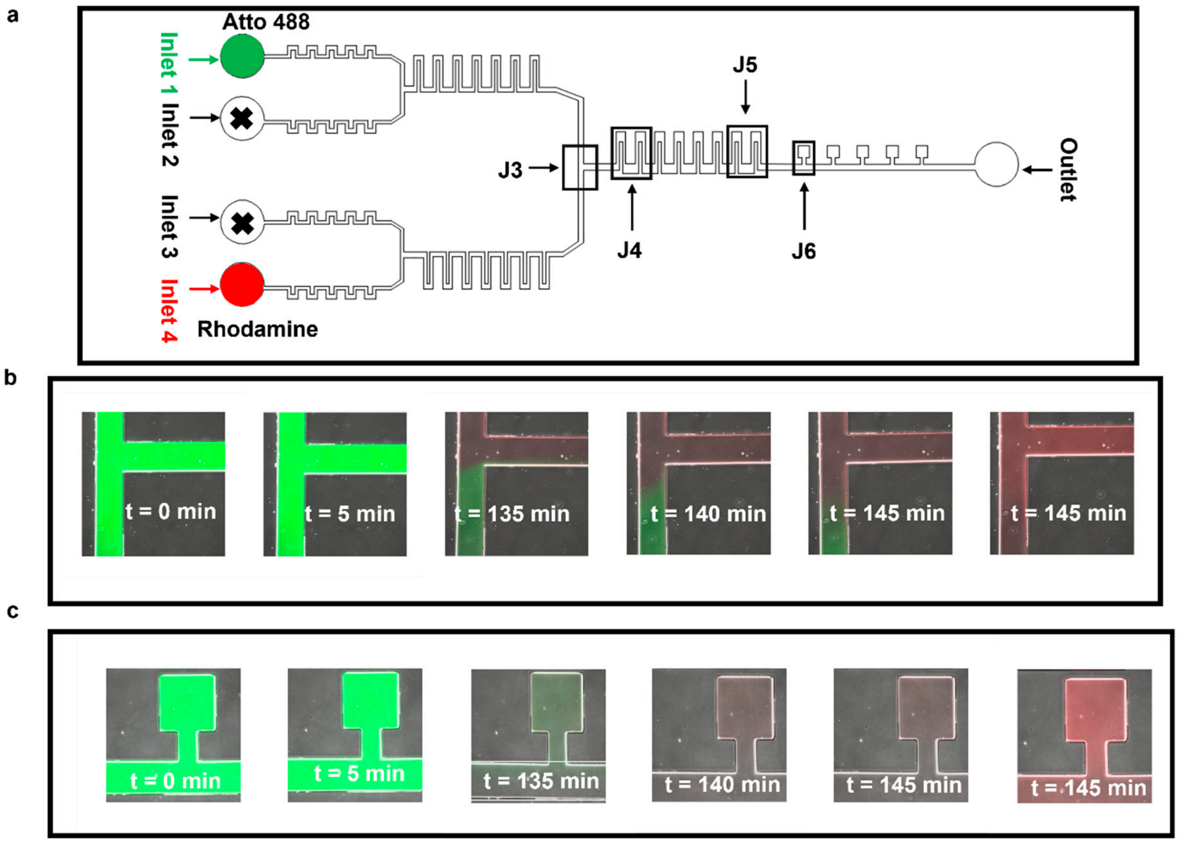

Figure S3. Media exchange experiments for extended time at 50 µL h^{-1}. (a) Microfluidic device design with highlighted parts. (b) Images of the junction indicated in (a). At the start of the experiment, we have only green dye, and at t = 135 min the inlet with green dye is switched off. The red dye starts to take over the junction, and at t=145 minutes it completely takes over the junction. (c) Images of the first cell culture chamber. At t=145 minutes, it completely takes over the cell culture chamber.
